# Hypothalamic endocannabinoids inversely correlate with the development of diet-induced obesity in male and female mice[S]

**DOI:** 10.1194/jlr.M092742

**Published:** 2019-05-28

**Authors:** Cristina Miralpeix, Anna Fosch, Josefina Casas, Miguel Baena, Laura Herrero, Dolors Serra, Rosalía Rodríguez-Rodríguez, Núria Casals

**Affiliations:** Basic Sciences Department, Faculty of Medicine and Health Sciences* Universitat Internacional de Catalunya, 08195 Sant Cugat del Vallès, Spain; Department on Biomedical Chemistry, Research Unit of BioActive Molecules† Institut de Química Avançada de Catalunya, 08034 Barcelona, Spain; Centro de Investigación Biomédica en Red de Enfermedades Hepáticas y Digestivas§ Instituto de Salud Carlos III, E-28029 Madrid, Spain; Centro de Investigación Biomédica en Red de Fisiopatología de la Obesidad y la Nutrición,** Instituto de Salud Carlos III, E-28029 Madrid, Spain; Department of Biochemistry and Physiology, School of Pharmacy†† Institut de Biomedicina de la Universitat de Barcelona, Universitat de Barcelona, E-08028 Barcelona, Spain

**Keywords:** hypothalamus, sexual dimorphism, brown adipose tissue

## Abstract

The endocannabinoid (eCB) system regulates energy homeostasis and is linked to obesity development. However, the exact dynamic and regulation of eCBs in the hypothalamus during obesity progression remain incompletely described and understood. Our study examined the time course of responses in two hypothalamic eCBs, 2-arachidonoylglycerol (2-AG) and arachidonoylethanolamine (AEA), in male and female mice during diet-induced obesity and explored the association of eCB levels with changes in brown adipose tissue (BAT) thermogenesis and body weight. We fed mice a high-fat diet (HFD), which induced a transient increase (substantial at 7 days) in hypothalamic eCBs, followed by a progressive decrease to basal levels with a long-term HFD. This transient rise at early stages of obesity is considered a physiologic compensatory response to BAT thermogenesis, which is activated by diet surplus. The eCB dynamic was sexually dimorphic: hypothalamic eCBs levels were higher in female mice, who became obese at later time points than males. The hypothalamic eCBs time course positively correlated with thermogenesis activation, but negatively matched body weight, leptinemia, and circulating eCB levels. Increased expression of eCB-synthetizing enzymes accompanied the transient hypothalamic eCB elevation. Icv injection of eCB did not promote BAT thermogenesis; however, administration of thermogenic molecules, such as central leptin or a peripheral β3-adrenoreceptor agonist, induced a significant increase in hypothalamic eCBs, suggesting a directional link from BAT thermogenesis to hypothalamic eCBs. This study contributes to the understanding of hypothalamic regulation of obesity.

The endocannabinoid (eCB) system is a highly conserved lipid-derived signaling system that plays a critical role in the control of energy homeostasis and body weight (1). The most well-known eCBs are 2-arachidonoylglycerol (2-AG) and *N*-arachidonoylethanolamine (anandamide; AEA). eCBs are synthetized on demand in the brain and peripheral tissues, where they can act in an autocrine or paracrine manner or be secreted to the bloodstream (2). In peripheral tissues, such as liver, fat, pancreas, and muscle, eCBs exert a wide range of metabolic effects, including the modulation of food digestion, energy expenditure, lipid storage, and glucose homeostasis (3, 4). The overall action of eCBs in the periphery favors energy intake and storage, promoting obesity development (1, 2). In humans, evidence demonstrates that circulating 2-AG and/or AEA levels are increased in people with obesity, and these levels specifically correlate with visceral fat mass, either in female or male patients (5, 6).

The eCB signaling is particularly critical in the brain, where it modulates neurotransmitter release and provides neuroprotection (7, 8). The eCB system is widely expressed in brain areas associated with the regulation of energy homeostasis, like the hypothalamus, the brainstem, and the cortico-limbic system (7). In the hypothalamus, 2-AG levels are increased in different genetic models of obesity: Zucker rats, *db*/*db* mice, or *ob*/*ob* mice (9). In line with these evidences, specific deletion of the main eCB receptor (CB1) in the hypothalamus resulted in increased energy expenditure and brown adipose tissue (BAT) thermogenesis, leading to a reduction in body weight, while food intake remained unchanged (10). These studies, together with others on genetic animal models (11–13), suggest that the activation of the eCB system in the hypothalamus leads to reduced energy expenditure and promotes obesity (1).

Despite these findings in genetic models, the exact dynamic of eCB levels and their modulation in the hypothalamus during diet-induced obesity (DIO), the model that best resembles human obesity, has been poorly explored, and the scarce results in response to a high-fat diet (HFD) are contradictory. For instance, hypothalamic 2-AG levels were increased in rats after long-term exposure to an HFD (24 weeks) (14), whereas they were not changed in another study performed with mice fed an HFD for 19 weeks (15). In both studies, AEA levels remained unchanged. Moreover, the time-course fluctuations and regulation of the eCB profile in the hypothalamus during obesity development remains incompletely described and understood.

In addition to this unsolved issue, recent evidence showed some differences in brain eCB levels between male and female mice after long-term administration of an HFD (16), in line with the sexually dimorphic brain response in obesity (17–19). However, eCB dynamics in the hypothalamus between male and female animals during DIO development have not been explored.

In the present study, we have analyzed hypothalamic 2-AG and AEA levels in male and female mice at different stages of DIO development. Our results demonstrate that 2-AG and AEA levels transitorily increase in both genders, with maximum levels at 7 days of HFD administration, followed by a gradual decline to levels similar to those observed in control groups. These changes positively correlate with BAT thermogenesis and inversely correlate with body-weight gain. Acute activation of BAT thermogenesis under different stimuli also increased eCB levels in the hypothalamus, indicating early rises in hypothalamic eCBs as a compensatory response to the increased thermogenesis. This is the first study revealing the exact dynamic of hypothalamic 2-AG and AEA during DIO development and its potential link to BAT activation in male and female mice.

## METHODS

### Animals, diets, and sample collection

Male and female C57BL/6J mice (8 weeks old) were used for the experiments. All animals were housed on a 12 h/12 h light/dark cycle in a temperature- and humidity-controlled room and were allowed free access to water and standard laboratory chow. Animals were placed on an HFD (60% kcal from fat; catalog no. D12492, Research Diets, New Brunswick, NJ) or standard diet (SD) (10% kcal from fat; catalog no. D12450B, Research Diets) for 7, 14, 28, 60, or 90 days. Diets were administered in two different sets of animals: *1*) mice fed an SD or HFD for 7, 14, or 28 days; and *2*) mice fed an SD or HFD for 60 or 90 days. At the end of the studies, animals were fasted for 1 h and euthanized by cervical dislocation, and tissues were collected for further molecular and biochemical analysis. For each animal, the hypothalamus and interscapular BAT were quickly removed, weighed, and stored at −80°C. Plasma was obtained after blood centrifugation. Tissue processing and analysis from both sets of animals were simultaneously performed. All animal procedures were performed in agreement with European guidelines (2010/63/EU) and approved by the University of Barcelona Local Ethical Committee (Procedure ref. 9659, Generalitat de Catalunya).

### Extraction and analysis of eCBs

Hypothalamic and plasma eCBs from both sets of animals were simultaneously extracted and analyzed as previously described by Gong et al. (20). Hypothalamus (6–8 mg wet tissue) was dounce-homogenized in 200 µl of ice-cooled deionized water containing a final concentration of 0.362 µM *N*-oleylethanolamine-d2 (OEA-d2) (Cayman Chemicals, Ann Arbor, MI) as internal standard for 2-AG and AEA calibration, 100 µM PMSF, and 0.01% butylated hydroxytoluene (BHT) (Sigma-Aldrich, Madrid, Spain), followed by a brief sonication. After that, half of the homogenized sample was kept at −20°C for protein quantification, and the other half was mixed with 400 µl of ethyl acetate/*n*-hexane (9:1, v/v) and vortexed for 5 min. After centrifugation (14,000 *g*, 4°C, 5 min), the upper layer was collected and evaporated using a nitrogen evaporator.

Plasma (25 µl) was mixed with 0.362 µM OEA-d2, 100 µM PMSF, and 0.01% BHT per sample. Lipid extraction was made with 250 µl of ethyl acetate/*n*-hexane (9:1, v/v), following the same protocol as for hypothalamus extraction.

eCB levels were analyzed by LC/MS/MS, following the protocol described by Gong et al. (20). Briefly, 2-AG, AEA, and OEA-d2 (Cayman Chemicals) were used for the calibration curve in an Acquity ultra-high-performance liquid chromatography (UPLC) (Waters, Singapore) system connected to a Xevo-TQS triple-quadropole Detector (Waters, Ireland) and controlled with Waters/Micromass MassLynx software. Chromatographic separation was performed on an Acquity UPLC BEH C_18_ column (1.7 µm particle size, 100 mm × 2.1 mm; Waters) with an isocratic mobile phase of formic acid 0.1% in water-acetonitrile (30:70, v/v). The flow rate was 0.3 ml/min. Detection was performed with an electrospray interface operating in the positive ion mode. The capillary voltage was set to 3.1 kV, the source temperature was 150°C, and the desolvation temperature was 500°C, acquiring the following selected reaction monitoring transitions: OAE-d2: 328.2–62.2 Da, cone voltage 50 V, and collision energy 10 eV; AEA: 348.2–62.2 Da, cone voltage 50 V, and collision energy 10 eV; and 2-AG: 379.2–287.1 Da, cone voltage 50 V, and collision energy 10 eV. eCB levels from each experimental group were normalized to its corresponding control group (SD).

### Analysis of plasma leptin levels

Plasma levels of leptin were determined by a mouse ELISA Kit (Crystal Chem, Zaandam, The Netherlands), following the manufacturer’s instructions.

### RNA preparation and quantitative RT-PCR

Total RNA was extracted from tissues using Trizol Reagent (Fisher Scientific, Madrid, Spain). Retrotranscription and quantitative RT-PCR was performed as previously described (21). SYBR Green or Taqman Gene Expression assay primers were used (IDT DNA Technologies, Leuven, Belgium) (supplemental Table S1). Relative mRNA levels were measured using the CFX96 Real-time System, C1000 Thermal Cycler (Bio-Rad, Madrid, Spain).

### BAT temperature measurements

Skin temperature surrounding BAT was visualized using a high-resolution infrared camera (FLIR Systems) and analyzed with a specific software package (FLIR-Tools-Software, FLIR, Kent, UK), as described (22, 23). Thermal images were acquired the day of euthanization.

### Icv administration of leptin and 2-AG + AEA combination

Two different experiments were performed:* 1*) icv leptin administration followed by evaluation of BAT thermogenesis and hypothalamic eCB levels; and *2*) icv administration of a mixture of eCBs that consists of combinations of 2-AG + AEA in different dosages to evaluate BAT thermogenesis. For both experiments, cannulae were stereotaxically implanted into the lateral cerebral ventricle under ketamine/xylazine ip anesthesia, as previously described (23). Lean mice fed a chow diet were individually caged and allowed to recover for 5 days before the experiment.

#### For leptin injection experiment.

On the experimental day, lean male mice received an icv administration of 2 µl of either vehicle (aqueous buffer containing 0.1% BSA) or leptin (0.1 µg/µl) (PeproTech, London, UK) 3 h after lights on (23). At 4 h after the injection, mice were euthanized by cervical dislocation, and hypothalamus and BAT were collected for further analysis.

#### For eCBs injection experiment.

On the experimental day, lean male mice received an icv administration of 2 µl of either vehicle (saline buffer containing 5% DMSO) or the eCB combination 2-AG + AEA (Cayman Chemicals) in two different dosages: dose 1 (0.5 µg of 2-AG + 0.005 µg of AEA) and dose 2 (2 µg of 2-AG + 0.02 µg of AEA). The doses of 2-AG and AEA were selected based on previous publications using icv or intrahypothalamic administration of these eCBs separately (24, 25) and also considering the different range of concentration found in the hypothalamic region under standard conditions (×50–100 higher concentrations of 2-AG compared with AEA).

### β3-adrenergic agonist-induced thermogenesis activation

Hypothalamic eCB levels were determined after adrenergic stimulation of BAT thermogenesis with the selective β3-adrenergic agonist CL 316,243 (Tocris Bioscience, Bristol, UK) in lean mice, as previously described (26, 27). At 4 h after ip injection of either CL 316,243 (10 mg/kg) or vehicle (aqueous buffer) (26), mice were euthanized by cervical dislocation, and hypothalamus and BAT were collected for further analysis.

### Statistical analysis

All results are expressed as mean ± SEM (n = 8–12). Analysis was conducted using GraphPad Prism 6 (GraphPad Software, La Jolla, CA). Statistical analysis was determined by one-way ANOVA when different diet groups within the same gender were compared and two-way ANOVA when groups between different genders were compared. In both cases, ANOVA was followed by a post hoc two-tailed Bonferroni test. For the analysis of the relation between variables, parameters were mathematically log-transformed to improve symmetry, and correlation was analyzed by Pearson’s test and lineal regression. *P* < 0.05 was considered significant.

## RESULTS

### Progression of DIO was delayed in female compared with male mice

**Fig. 1.**
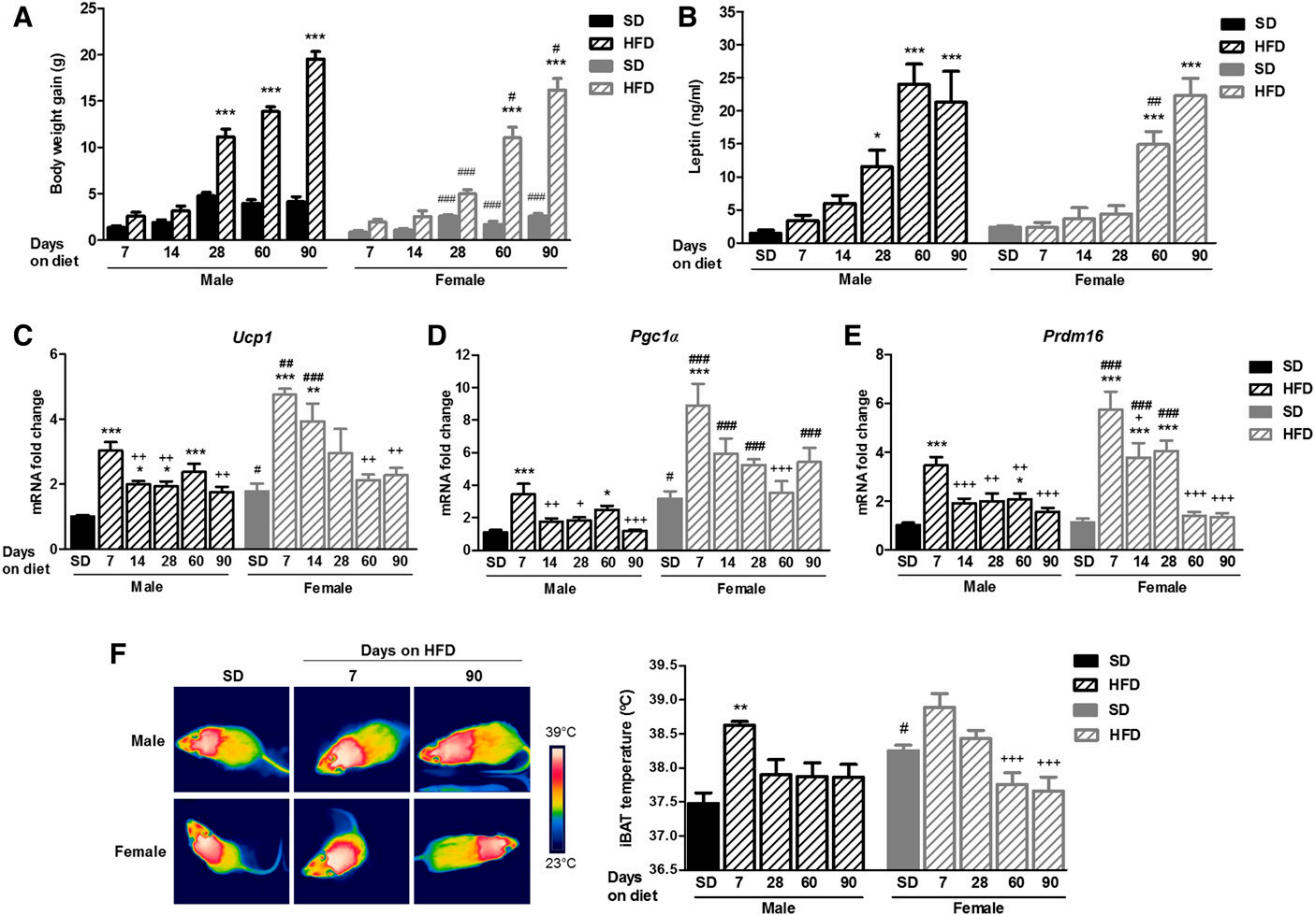
DIO development of male and female mice fed an SD or HFD for 90 days. A: Body weight gain. B: Plasmatic levels of leptin. C–E: Relative mRNA expression of the thermogenic markers UCP1 (C), PGC1α (D), and PRDM16 (E) in BAT of male and female mice fed an SD or HFD for 90 days (n = 8–10). F: Representative infrared thermal images and quantification of interscapular temperature adjacent to the BAT depot of male and female mice fed an SD or HFD for 7, 28, 60, and 90 days (n = 5). Statistical significance was determined by ANOVA and Bonferroni posttest. Error bars represent SEM. *P < 0.05; **P < 0.01; ***P < 0.001 versus its corresponding SD; ^#^*P* < 0.05; ^##^*P* < 0.01; ^###^*P* < 0.001 versus male under the same diet conditions; ^+^*P* < 0.05; ^++^*P* < 0.01; ^+++^*P* < 0.001 versus same gender fed an HFD for 7 days.

Age-matched male and female mice were fed an SD or an HFD for 90 days. During this period, progression of body weight gain, plasmatic levels of leptin, food, and caloric intake were evaluated (**Fig. 1**, supplemental Figs. S1, S2). Males fed an HFD for a period of administration equal to or longer than 28 days gained significantly more weight than controls (Fig. 1A, supplemental Fig. S1). In female mice, a significant increase in body weight gain was not appreciated until 60 days of administration of an HFD compared with an SD (Fig. 1A, supplemental Fig. S1). In line with these results, plasmatic levels of leptin were significantly increased in male mice after 28 days of HFD feeding compared with control diet, whereas female mice did not show hyperleptinemia until 60 days of HFD administration, and leptin levels were considerably lower at this point than those observed in male mice (Fig. 1B). These data show that male mice became obese and hyperleptinemic at earlier time points of HFD feeding than females, and the final body weights remained higher in comparison to female mice (Fig. 1A, B, supplemental Fig. S1).

As expected, total caloric intake in male and female mice was increased when the animals were fed an HFD, whereas food intake was decreased in HFD animals of both sexes compared with SD (supplemental Fig. S2). In addition, both caloric and food intake was significantly lower in female mice fed an SD or HFD in comparison to diet-matched male animals (supplemental Fig. S2).

### Short-term administration of an HFD induced a transient increase of both thermogenesis activation and hypothalamic eCB levels in male and female mice

The induction of thermogenesis in the interscapular BAT of male and female mice was analyzed at different time points during 90 days of HFD feeding, compared with SD (Fig. 1C–F). A substantial activation peak was reached in gene expression of thermogenic markers (Fig. 1C–E) and interscapular temperature (Fig. 1F) in the BAT of male and female mice after 7–14 days of an HFD when compared with an SD. Longer administration periods of HFD (from 28 days onwards) were not able to induce such a considerable activation of BAT thermogenesis (Fig. 1C–F). The activation of gene expression of thermogenic markers was higher in the BAT of female compared with male mice, particularly after 7, 14, and 28 days of an HFD (Fig. 1C–E). In addition, an increased basal expression of specific thermogenic genes (Fig. 1C, D) and basal interscapular BAT temperature (Fig. 1F) was appreciated in female mice in comparison to male mice, as described previously in the literature (28, 29).

**Fig. 2.**
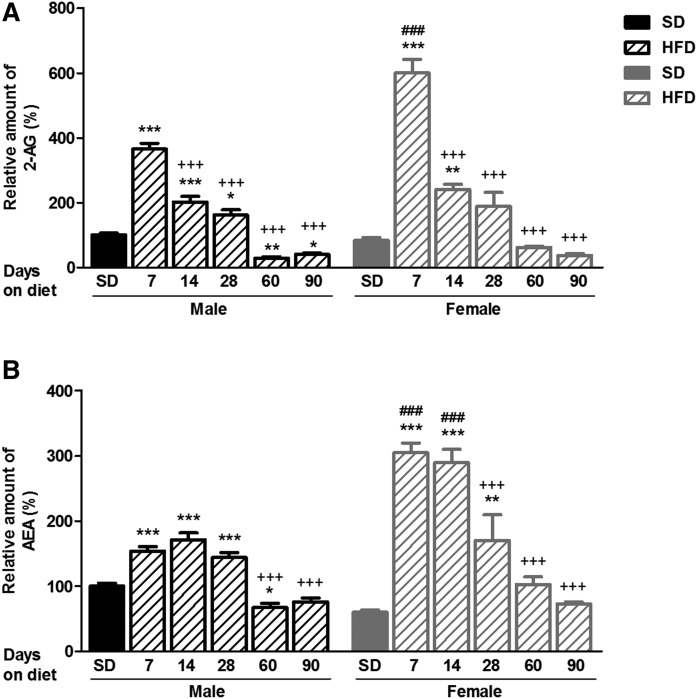
Time profile of hypothalamic eCB levels during the development of DIO in male and female mice. A: Relative amount of 2-AG to male SD. B: Relative amount of AEA to male SD. Statistical significance was determined by ANOVA and Bonferroni posttest. Error bars represent SEM (n = 8–10). *P < 0.05; **P < 0.01; ***P < 0.001 versus its corresponding SD; ^###^*P* < 0.001 versus male under the same diet conditions; ^+++^*P* < 0.001 versus same gender fed an HFD for 7 days.

Analysis of hypothalamic eCB levels also revealed a pronounced transitory increase after short-term administration of an HFD (**Fig. 2**). Hypothalamic 2-AG levels were significantly increased after 7 days of HFD feeding in both male and female mice, and these values were progressively attenuated, reaching basal levels in a time-dependent manner (Fig. 2A). AEA levels in the hypothalamus of male and female mice remained elevated during 7–28 days of HFD feeding, whereas they were significantly reduced after longer exposure to an HFD (Fig. 2B). Interestingly, transitory hypothalamic increases in 2-AG and AEA were substantially higher in female than in male mice, and decreases were more pronounced in female than in male mice (Fig. 2). Hypothalamic concentrations of 2-AG and AEA (ng of eCB/mg of tissue) derived from the two different sets of animals during dietary administration are shown in supplemental Table S2.

In summary, short-term exposure to an HFD induced a transitory activation of BAT thermogenesis and a transient increase in hypothalamic 2-AG and AEA in mice, and these responses were sexually dimorphic (Fig. 1, 2).

### Hypothalamic eCB levels correlated with body weight gain, leptinemia, and brown fat thermogenesis

**Fig. 3.**
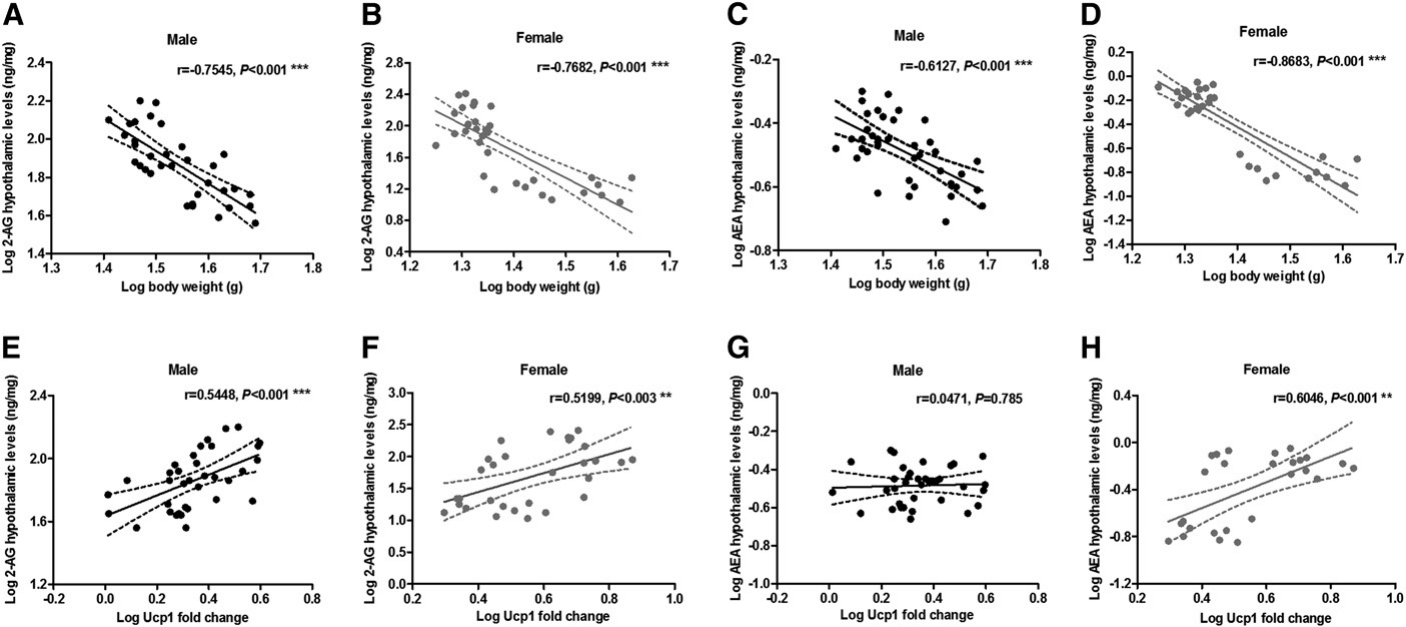
Correlation between hypothalamic eCBs and body weight or UCP1 mRNA expression in BAT. Hypothalamic 2-AG levels negatively correlate with body weight at time of euthanization in both male (A) and female (B) mice. Hypothalamic AEA levels negatively correlate with body weight at time of euthanization in both male (C) and female (D) mice. Hypothalamic 2-AG levels positively correlate with UCP1 mRNA expression in BAT of male (E) and female (F) mice. Hypothalamic AEA levels do not correlate with UCP1 mRNA expression in BAT of male mice (G), but they positively correlate in BAT of female mice (H). Statistical significance and correlation was determined by Pearson correlation coefficients (*xy* values = 30–40). **P < 0.01; ***P < 0.001.

First, we analyzed the relationship between hypothalamic eCB levels and body weight gain or plasma leptin levels in the animals fed the experimental diets. 2-AG and AEA levels in the hypothalamus showed a negative correlation with both body weight gain (**Fig. 3A**–D) and leptinemia (supplemental Fig. S3) in male and female mice. Then, evaluation of the relationship between eCBs in the hypothalamus and the mRNA expression levels of BAT thermogenesis activation revealed that hypothalamic 2-AG and AEA levels positively correlated with mRNA levels of Ucp1, Pgc1α, and Prdm16 in the BAT of female mice (Fig. 3F, H, supplemental Fig. S4). In male mice, this correlation was only significantly appreciated when analyzing 2-AG levels (Fig. 3E, G, supplemental Fig. S4).

These results indicate a negative association between eCB levels in the hypothalamus and obesity progression in male and female mice, but a positive association of hypothalamic eCBs with thermogenic activation in response to an HFD, particularly evidenced in female mice.

### Plasmatic eCBs increased after longer administration periods of an HFD and showed a negative correlation to hypothalamic eCB levels

**Fig. 4.**
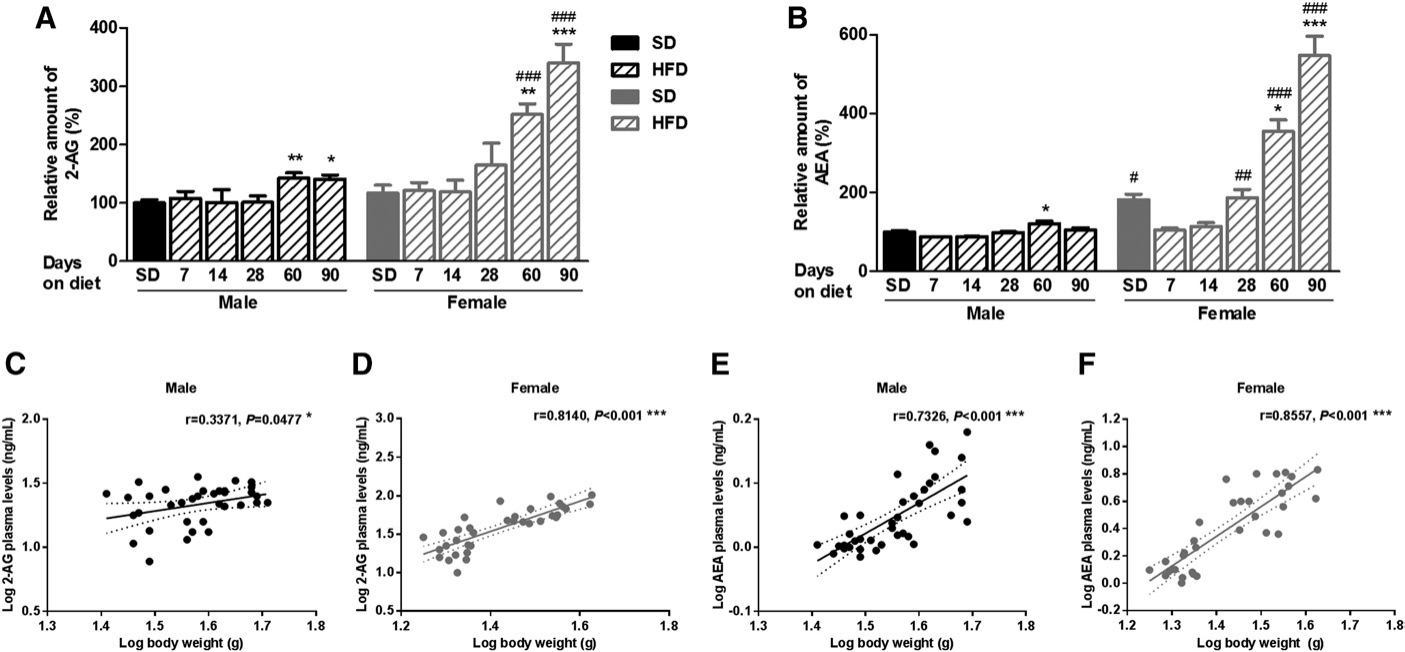
Time profile of plasmatic eCBs levels during the development of DIO and correlation to body weight in male and female mice. A: Relative amount of 2-AG compared with values found in male SD. B: Relative amount of AEA compared with values found in male SD. Plasmatic 2-AG levels positively correlate with body weight at time of euthanization in both male (C) and female (D) mice. Plasmatic AEA levels positively correlate with body weight at time of euthanization in both male (E) and female (F) mice. Statistical significance was determined by ANOVA and Bonferroni posttest, and correlation was determined by Pearson correlation coefficients (*xy* values = 33–39). Error bars represent SEM (n = 8–10). *P < 0.05; **P < 0.01; ***P < 0.001 versus its corresponding SD; ^#^*P* < 0.05; ^##^*P* < 0.01; ^###^*P* < 0.001 versus male mice under the same diet conditions.

In contrast to the hypothalamus, plasmatic levels of 2-AG and AEA remained unchanged after short-term administration of an HFD compared with SD in both male and female mice (**Fig. 4A**, B). However, exposure to an HFD for 60 or 90 days revealed an increase in plasmatic 2-AG in both male and female mice (Fig. 4A) and a substantial increase in plasmatic AEA, particularly observed in female mice (Fig. 4B). In addition, 2-AG and AEA levels in plasma evidenced a positive correlation with body weight gain in both male and female mice (Fig. 4C–F). Analysis of the relationship between plasmatic and hypothalamic eCB levels revealed that plasmatic eCBs negatively correlated with those levels in the hypothalamus of male and female mice fed an HFD (supplemental Fig. S5). Plasmatic concentrations of 2-AG and AEA (ng of eCB/ml of plasma) derived from the two different sets of animals during dietary administration are shown in supplemental Table S3.

### Short-term HFD feeding increased gene expression of eCB synthesis enzymes in the hypothalamus

**Fig. 5.**
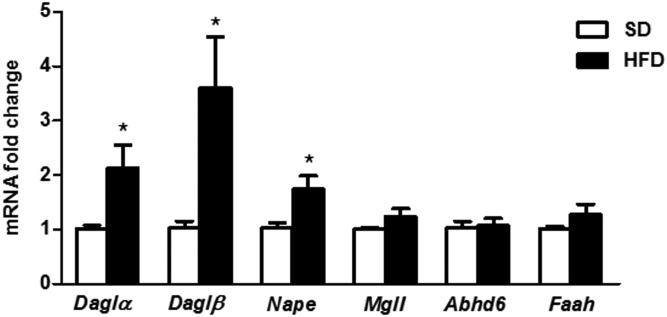
Relative mRNA expression of 2-AG and AEA synthesis and degradation enzymes in hypothalamus of male mice fed an SD or HFD for 7 days. Statistical significance was determined by ANOVA and Bonferroni posttest. Error bars represent SEM (n = 5–8). *P < 0.05 versus SD.

We analyzed gene expression of the enzymes responsible for the synthesis (*Daglα*, *Daglβ*, and *Nape*) and degradation (*Mgll*, *Abhd6*, and *Faah*) of 2-AG and AEA in the hypothalamus of mice fed an SD or HFD for 7 days. This analysis revealed a significant increase in the expression of the hypothalamic enzymes synthetizing 2-AG and AEA after short-term HFD administration, whereas this increase was not evidenced in the expression of degradation enzymes (**Fig. 5**). Overall, this result shows that the transient increase in hypothalamic 2-AG and AEA over 7 days of HFD feeding concurs with an enhanced expression of synthesis enzymes in the hypothalamus.

### Acute leptin and a β3-adrenergic agonist both induced a thermogenic response with an increase in hypothalamic eCB levels

**Fig. 6.**
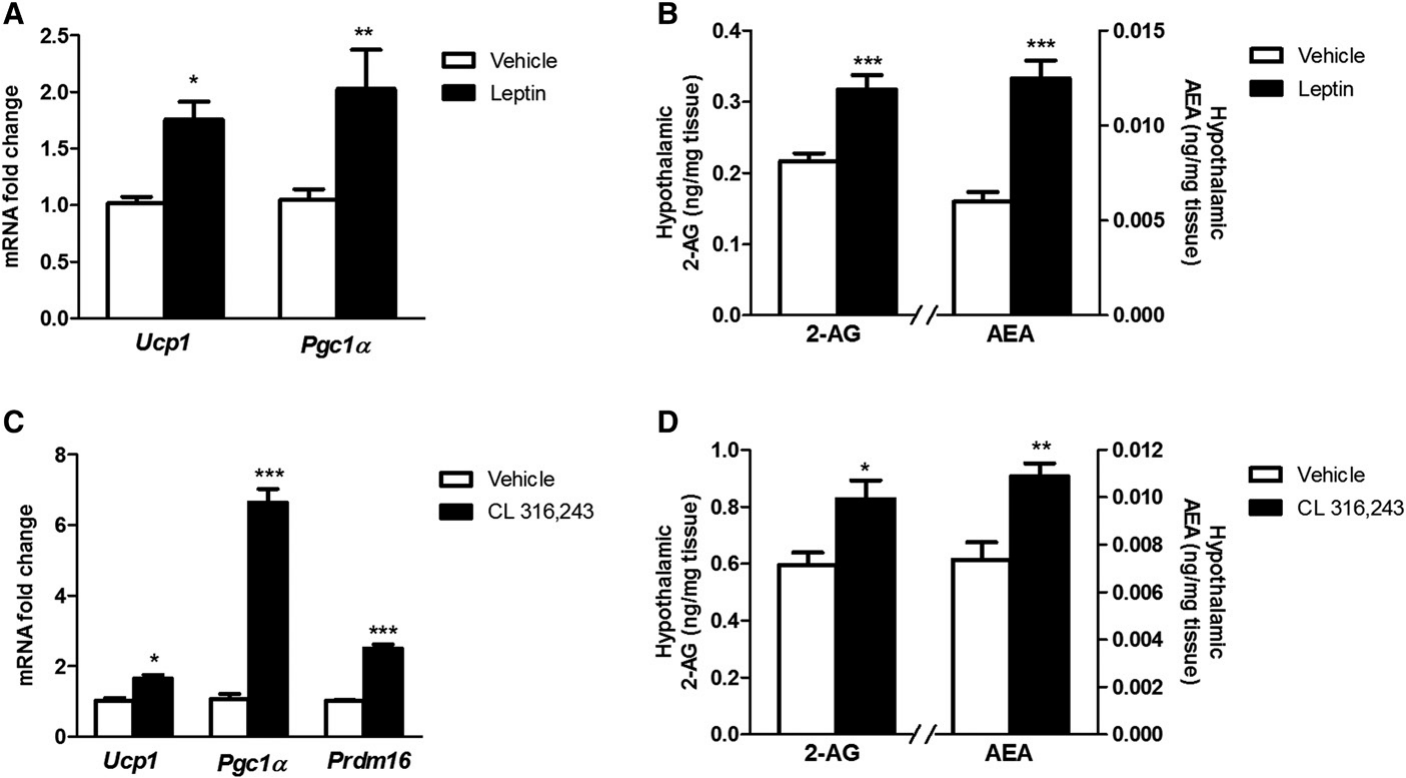
Hypothalamic eCB levels and BAT thermogenesis in response to acute icv leptin (A, B) or to ip injection of the β3-adrenoreceptor agonist CL 316,243 (C, D) in lean mice fed a chow diet. Relative mRNA expression of the thermogenic markers in BAT samples after 4 h of icv leptin (A) or ip CL 316,243 administration (C). Concentration of 2-AG and AEA in the hypothalamus of mice after 4 h of icv leptin (B) or ip CL 316,243 administration (D). Statistical significance was determined by ANOVA and Bonferroni posttest. Error bars represent SEM (n = 8–12). *P < 0.05; **P < 0.01; ***P < 0.001 versus vehicle.

We decided to measure the levels of hypothalamic eCBs in another condition well known to activate BAT thermogenesis, such as central leptin administration and ip administration of a β3-adrenergic agonist. In agreement with our previous results (23), 4 h of leptin icv increased gene expression of thermogenic markers in BAT (**Fig. 6A**). Under these experimental conditions, hypothalamus of the same animals evidenced a significant increase in both 2-AG and AEA concentrations (Fig. 6B).

To compare the effects of central leptin versus peripheral adrenergic activation of BAT, mice received ip injection of the potent and selective β3-adrenergic agonist CL 316,243, an agonist that has demonstrated minimal access to the brain after peripheral injection (30). In line with leptin experiment, CL 316,243 induced adrenergic activation of BAT thermogenesis marker genes (Fig. 6C), with a substantial increase in both 2-AG and AEA in the hypothalamus (Fig. 6D), revealing an association between hypothalamic eCBs and thermogenesis activation.

### Acute central administration of 2-AG + AEA did not induce BAT thermogenesis

**Fig. 7.**
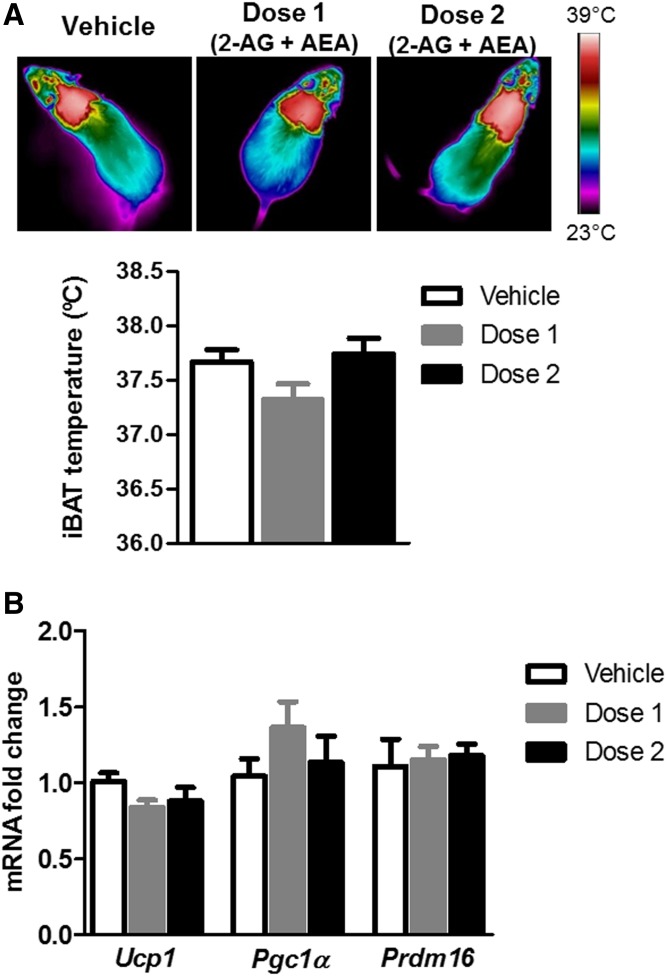
BAT thermogenesis in response to acute icv injection of the eCBs 2-AG and AEA to lean mice, in two different dosages: dose 1 (0.5 µg 2-AG + 0.005 µg AEA) and dose 2 (2 µg 2-AG + 0.02 µg AEA). Representative infrared thermal images and the corresponding quantification of interscapular temperature adjacent to the BAT depot (iBAT) (A) and relative mRNA expression of the thermogenic marker UCP1 (B) in BAT samples of mice after 4 h of icv injection of 2-AG + AEA in two different dosages. Statistical significance was determined by ANOVA and Bonferroni posttest. Error bars represent SEM (n = 8–10).

Icv administration of the mixture of the eCBs, 2-AG + AEA, in two different doses for 4 h, was not able to induce a significant alteration of either interscapular BAT temperature (**Fig. 7A**) or gene expression levels of thermogenic markers in BAT (Fig. 7B). Food intake was not significantly altered in response to icv injection of the doses of these eCBs (data not shown), in agreement with a previous publication (25).

## DISCUSSION

Despite the well-established function of the eCB system on energy homeostasis, our knowledge on its exact dynamic and regulation under dietary conditions leading to obesity and associated complications is still limited. Our current study presents for the first time the temporal profile of hypothalamic eCB changes during the development of DIO and its association with BAT thermogenesis activation and leptin response in male and female mice. This is an intriguing finding, considering the very few and contradictory evidence that exists in the literature on hypothalamic eCBs in response to an HFD, its relation to BAT activation, and the potential contribution of this association to sexual dimorphism in obesity.

One of the most remarkable results in our study was the transitory and substantial increase in hypothalamic eCBs after short-term administration of an HFD in both male and female mice, while these levels were progressively attenuated under long-term exposure to this diet. The transient increase was particularly pronounced on 2-AG levels after 7 days of HFD feeding (four to six times higher than the basal levels). At this time point, the early rise on eCB levels was sustained by a significant increase in the hypothalamic expression of the enzymes responsible for the synthesis of 2-AG and AEA, without significant alterations in degrading enzyme expression.

It has been reported that eCB levels become deregulated in the hypothalamus during obesity (7, 9). Di Marzo et al. (9) were the first detecting elevated levels of 2-AG in genetically obese Zucker rats and *ob*/*ob* mice and of both 2-AG and AEA in the hypothalamus of *db*/*db* mice, compared with lean controls. Because those genetic models are either leptin-deficient or express mutated forms of the leptin receptor, the increased eCB levels in the hypothalamus were suggested as an additional component of leptin-sensitive regulatory mechanisms (9). Studies on DIO animals, the model that best resembles human obesity, are scarce and controverted in terms of hypothalamic eCB changes. Moreover, some of these investigations are limited to their analysis after long-term HFD administration (19 weeks onward) and thereby when obesity has been already established (14, 15). Our current data show that hypothalamic 2-AG and AEA levels are not increased when DIO is established (at 60 or 90 days of an HFD), but they are at earlier stages (7–28 days) of high-fat feeding, in contrast to that observed in plasma. These data indicate that the eCB dynamic in the hypothalamus is different in genetic animal models of obesity compared with diet-induced obese models. Furthermore, this is the first time that a negative association between hypothalamic eCBs and body weight during DIO development is demonstrated.

Our group has recently reported that the robust activation peak of BAT thermogenesis observed under 7 days of HFD feeding matches with a transient preservation of normal body weight, adiposity, and leptinemia in the initial phases of DIO (23). The fact that the transitory increase in hypothalamic eCBs after short-term HFD administration found in the present investigation correlated with the activation peak of BAT thermogenesis led us to propose this early rise in hypothalamic eCBs as a physiological compensatory response (found in the hypothalamus, but not in plasma) to BAT thermogenesis activation triggered by diet surplus. These data also suggest the existence of a cross-talk between BAT and hypothalamic eCBs in the initial stages of obesity.

The link between the increase in hypothalamic 2-AG and AEA levels and BAT thermogenesis activation was further evaluated by measuring hypothalamic eCBs following acute thermogenic activation by different stimuli. Acute administration of either central leptin or ip injection of CL 316,243, a selective and peripherally restricted β3-adrenergic agonist, induced BAT thermogenesis activation with a substantial increase in hypothalamic eCB levels. This is the first study revealing that acute stimulation of BAT enhances eCB levels in the hypothalamus. In relation to these findings, it was recently demonstrated that in vivo stimulation of β3-adrenoreceptors in brown adipocytes by CL 316,243 (acutely or chronically injected) elevated the levels of the eCBs, 2-AG and AEA, in BAT and were suggested to act as local autocrine negative-feedback regulators (27). Our assumption that the hypothalamic eCB elevation is a compensatory response, and not a cause, to BAT thermogenesis activation was also supported by the absence of stimulation of thermogenesis under icv administration of different doses of the combination of 2-AG + AEA. The lack of BAT activation by central eCB injection, at least with the selected dosages, suggests the unidirectional link from BAT to hypothalamic eCBs. However, further research is needed to unravel the molecular mechanisms by which BAT is possibly signaling the hypothalamic eCB system, particularly in early stages of DIO development.

Therefore, the present investigation shows evidence that, in early stages of obesity, when BAT thermogenesis is more active, there is a higher increase in hypothalamic eCBs, as a physiological compensatory response to BAT activation. However, when DIO is already established, the decline of thermogenesis activation is accompanied by a decrease in hypothalamic eCB levels. Further investigation will be needed to study the specific role of hypothalamic eCBs in response to BAT activation.

Interestingly, we can find in the literature other examples of short-term changes in the hypothalamus in response to nutritional surplus suggested to be compensatory mechanisms in obesity (31–33). These changes have also been described as processes preceding insulin/leptin sensitivity disruption and inflammation in peripheral tissues and therefore promoting positive energy balance (34, 35). Therefore, we might also understand the transitory increase in hypothalamic eCBs as an early indicator to precede leptin resistance and peripheral obesity.

Despite the discrepancy on central eCBs during DIO, the dysregulation of circulating eCB levels in metabolic diseases have been widely investigated (1–3). To date, these data are more conclusive, and the results on plasmatic eCBs in rodents are similar to those observed in humans (1, 36–38). Circulating eCBs positively correlate with markers of obesity and metabolic disorders, such as BMI, waist circumference, visceral fat mass, and insulin resistance (5, 6, 37, 39, 40). Our data on circulating eCBs agree with those previously reported—that is, plasmatic 2-AG and AEA levels were increased, particularly in female mice, after long-term exposure to an HFD, but not at early stages of DIO. Interestingly, these results negatively correlated with those concentrations in the hypothalamus. In line with these evidences, Kuipers et al. (41) recently demonstrated that there is a link between eCB metabolism in adipose tissues and plasmatic eCBs during DIO development. They showed that long-term HFD feeding increases circulating eCBs, accompanied by increased synthesis enzymes in adipose tissue (particularly BAT) of DIO mice (41). The authors also suggest that adipose tissues are likely important organs that release 2-AG and AEA levels in HFD-induced obesity (41).

An important finding in the current investigation was the difference in eCB levels observed depending on the gender. Relevant differences were appreciated between male and female mice, particularly when analyzing the transient increase of hypothalamic eCBs and the peak of activation of thermogenesis after a short-term HFD, which were substantially higher in females. In addition, obesity progression was delayed and less severe in females, suggesting a sexual dimorphism in hypothalamic eCB systems that could determine obesity progression. The relationship of central eCB systems and sexual dimorphism in obesity has been poorly explored (16). Recent findings on hypothalamic dimorphism in fatty acid concentration, chain length, and saturation, in response to HFD feeding were associated with protection to obesity and cardiovascular diseases in female compared with male mice (17, 19). Our data are the first comparing the dynamic of hypothalamic eCBs between male and female mice during DIO development. These results are therefore contributing to elucidate the relevance of central lipid metabolism in the sexual dimorphism in obesity.

Overall, this is the first study revealing the exact dynamic of hypothalamic eCBs during the development of obesity in DIO models, and these temporal changes correlated positively to BAT thermogenesis and negatively to circulating eCB, leptin, and body weight. Our data evidence a transitory elevation in hypothalamic eCBs after short-term HFD feeding accompanied by increased expression of 2-AG and AEA synthesis enzymes, understood as a physiological compensatory response to BAT thermogenesis activation triggered by diet surplus. The link between hypothalamic eCBs and BAT thermogenesis activation was also supported by a substantial upregulation of eCB in the hypothalamus following acute thermogenic activation by central leptin or peripheral β3-adrenergic stimulation. Our findings could add significant insight into the understanding of the hypothalamic mechanisms regulating obesity progression and its relationship to BAT function.

## Supplementary Material

Supplemental Data
