# Supplementary material for: Hypothalamic endocannabinoids inversely correlate with the development of diet-induced obesity in male and female mice
Source: J Lipid Res. 2019 May 28;60(7):1260–9. doi: 10.1194/jlr.M092742 (PMC6602126; doi:10.1194/jlr.M092742)
Supplement: Supplemental Data [file supp_60_7_1260__index.html]

Hypothalamic endocannabinoids inversely correlate with the development of diet-induced obesity in male and female mice — Hypothalamic endocannabinoids inversely correlate with the development of diet-induced obesity in male and female mice — Supplemental Data 

# Hypothalamic endocannabinoids inversely correlate with the development of diet-induced obesity in male and female mice

## Supplemental Data

- Supplemental figure S1 (.docx, 57 KB) - Body weight at time of sacrifice.
- Supplemental figure S2 (.docx, 39 KB) - Total caloric (A) and food intake (B) of male and female mice fed a standard diet (SD) or a high fat diet (HFD) for 90 days
- Supplemental figure S3 (.docx, 39 KB) - Correlation between hypothalamic endocannabinoids and plasmatic leptin levels.
- Supplemental figure S4 (.docx, 120 KB) - Correlation between hypothalamic endocannabinoids and Prdm16 or Pgc1?????????????????????????????????????? mRNA expression in BAT
- Supplemental figure S5 (.docx, 105 KB) - Correlation between hypothalamic and plasmatic endocannabinoids in male and female mice
- Supplemental Table S1 (.docx, 14 KB) - Quantitative real-time PCR primers
- Supplemental Table S2 (.docx, 15 KB) - Hypothalamic endocannabinoids levels
- Supplemental Table S3 (.docx, 15 KB) - Plasmatic endocannabinoids levels
