# Supplementary material for: Hypothalamic endocannabinoids inversely correlate with the development of diet-induced obesity in male and female mice
Source: J Lipid Res. 2019 May 28;60(7):1260–9. doi: 10.1194/jlr.M092742 (PMC6602126; doi:10.1194/jlr.M092742)
Supplement: Supplemental Data [file 10.1194_M092742_jlr.M092742-1.docx]

**
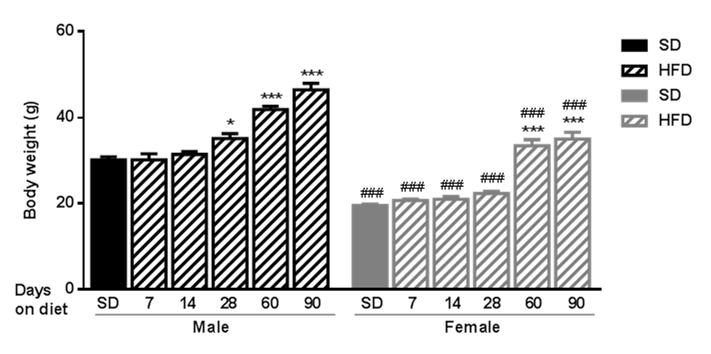
**

**Supplemental Fig. S.1.** Body weight at time of sacrifice. Statistical significance was determined by ANOVA and Bonferroni post-test. Error bars represent SEM (n=8-10). **P*<0.05, ****P*<0.001 *versus* its corresponding SD; ^###^*P* <0.001 *versus* male under the same diet conditions.
