# Supplementary material for: Hypothalamic endocannabinoids inversely correlate with the development of diet-induced obesity in male and female mice
Source: J Lipid Res. 2019 May 28;60(7):1260–9. doi: 10.1194/jlr.M092742 (PMC6602126; doi:10.1194/jlr.M092742)
Supplement: Supplemental Data [file 10.1194_M092742_jlr.M092742-2.docx]

**
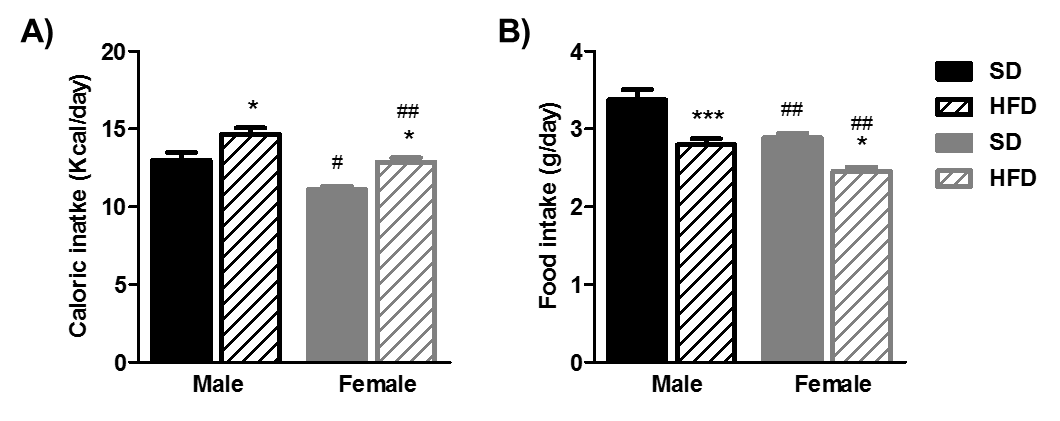
**

**Supplemental Fig. S.2.** Total caloric (A) and food intake (B) of male and female mice fed a standard diet (SD) or a high fat diet (HFD) for 90 days. Statistical significance was determined by ANOVA and Bonferroni post-test. Error bars represent SEM (n=8-10). **P* < 0.05, ****P* < 0.001 versus its corresponding SD; #*P* < 0.05, ##*P* < 0.01 versus male under the same diet conditions.
