# Supplementary material for: Hypothalamic endocannabinoids inversely correlate with the development of diet-induced obesity in male and female mice
Source: J Lipid Res. 2019 May 28;60(7):1260–9. doi: 10.1194/jlr.M092742 (PMC6602126; doi:10.1194/jlr.M092742)
Supplement: Supplemental Data [file 10.1194_M092742_jlr.M092742-4.docx]

**
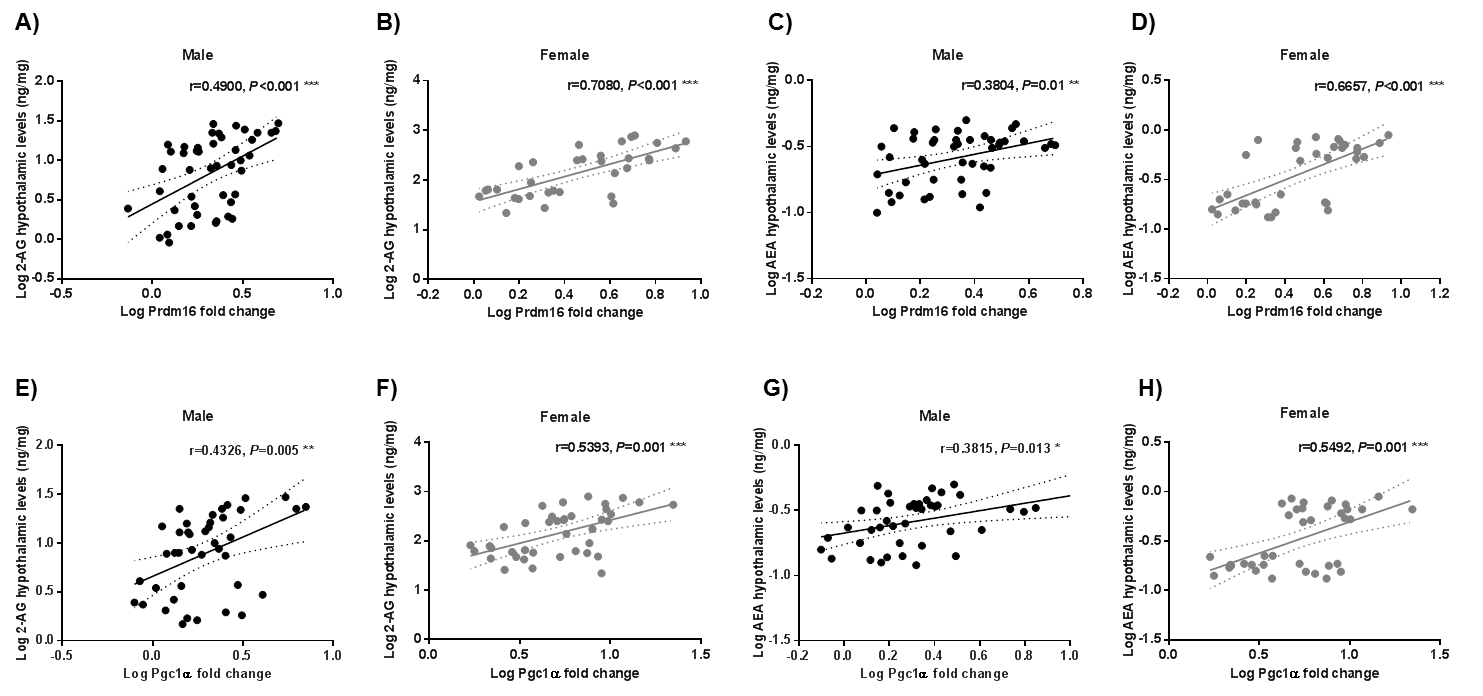
**

**Supplemental Fig. S.4.** Correlation between hypothalamic endocannabinoids and Prdm16 or Pgc1α mRNA expression in BAT. (A-B) Hypothalamic 2-AG levels positively correlate with Prdm16 mRNA expression in BAT in both male (A) and female (B) mice. (C-D) Hypothalamic AEA levels positively correlate with Prdm16 mRNA expression in BAT in both male (C) and female (D) mice. (E-F) Hypothalamic 2-AG levels correlate with Pgc1α mRNA expression in BAT in both male (E) and female mice (F). (G-H) Hypothalamic AEA levels correlate with Pgc1α mRNA expression in BAT in both male (G) and female mice (H). Statistical significance and correlation was determined by Pearson correlation coefficient (XY values=30-36).
