# Supplementary material for: Hypothalamic endocannabinoids inversely correlate with the development of diet-induced obesity in male and female mice
Source: J Lipid Res. 2019 May 28;60(7):1260–9. doi: 10.1194/jlr.M092742 (PMC6602126; doi:10.1194/jlr.M092742)
Supplement: Supplemental Data [file 10.1194_M092742_jlr.M092742-5.docx]

**
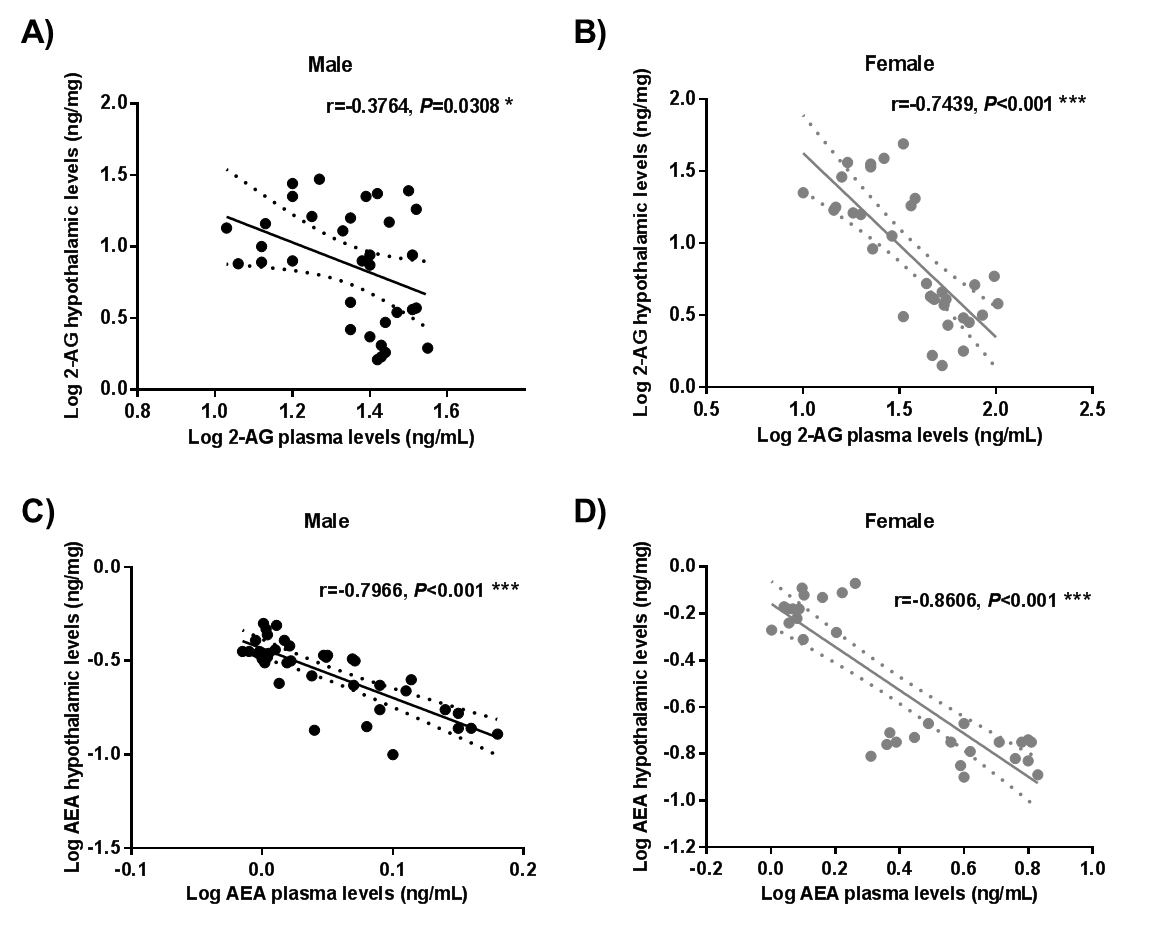
**

**Supplemental Fig. S.5.** Correlation between hypothalamic and plasmatic endocannabinoids in male and female mice. (A-B) Hypothalamic 2-AG levels negatively correlate with plasmatic 2-AG levels in both male (A) and female (B) mice. (C-D) Hypothalamic AEA levels negatively correlate with plasmatic AEA levels in both male (C) and female (D) mice. Statistical significance and correlation was determined by Pearson correlation coefficients (XY values=26-36).
