# Supplementary material for: Hypothalamic endocannabinoids inversely correlate with the development of diet-induced obesity in male and female mice
Source: J Lipid Res. 2019 May 28;60(7):1260–9. doi: 10.1194/jlr.M092742 (PMC6602126; doi:10.1194/jlr.M092742)
Supplement: Supplemental Data [file 10.1194_M092742_jlr.M092742-6.docx]

**Supplemental Table S.1.** Quantitative real-time PCR primers (SyBr or Taqman).

| **Gene (SyBr)** | **Sequence** |
| --- | --- |
| *Abhd6* | Fw: 5'-AGACCAGGTGCTTGATGT  Rv: 5'-CTCTCCATCACTACCGAAT |
| *Daglα* | Fw: 5'-TATCTTCCTCTTCCTGCT  Rv: 5'-CCATTTCGGCAATCATAC |
| *Daglβ* | Fw: 5'-GGGTCTTTTGAGCTGTTC  Rv: 5'-AAGGAGGACTATCAGGTA |
| *Faah* | Fw: 5'-CAGCTACAAGGGCCATGCT  Rv: 5'-TTCCACGGGTTCATGGTCTG |
| *Gapdh* | Fw: 5'-TCCACTTTGCCACTGCA  Rv: 5'-GAGACGGCCGCATCTTCTT |
| *MglI* | Fw: 5'-CCCAGTGGCACACCCAAG  Rv: 5'-TAACGGCCACAGTGTTCCC |
| *Nape* | Fw: 5'-AAAACATCTCCATCCCGAA  Rv: 5'-CGTCCATTTCCACCATCA |
| *Pgc1α* | Fw: 5'-GAAAGGGCCAAACAGAGAGA  Rv: 5'- GTAAATCACACGGCGCTCTT |
| *Prdm16* | Fw: 5'-CCTAAGGTGTGCCCAGCA  Rv: 5'-CACCTTCCGCTTTTCTACCC |
| **Gene (Taqman)** | **Sequence** |
| *Ucp1* | Fw: 5’-CACACCTCCAGTCATTAAGCC  Rv:5’-CAAATCAGCTTTGCCTCACTC  Assay name: Mm.PT.58.7088262 |
