# Supplementary material for: Hypothalamic endocannabinoids inversely correlate with the development of diet-induced obesity in male and female mice
Source: J Lipid Res. 2019 May 28;60(7):1260–9. doi: 10.1194/jlr.M092742 (PMC6602126; doi:10.1194/jlr.M092742)
Supplement: Supplemental Data [file 10.1194_M092742_jlr.M092742-7.docx]

**Supplemental Table S.2.** Hypothalamic endocannabinoids levels (ng/mg tissue).

|  | | **2-AG** | | **AEA** | | |  |
| --- | --- | --- | --- | --- | --- | --- | --- |
| ***Days on diet*** | | ***Male*** | ***Female*** | | ***Male*** | ***Female*** | |
| **SD** |  | 2.7 ± 0.3 | 1.6 ± 0.5 | | 0.013 ± 0.001 | 0.008 ± 0.001 | |
| **HFD** | **7** | 8.6 ± 1.4 ^a^ | 11.5 ± 1.4 ^a, b^ | | 0.023 ± 0.005 ^a^ | 0.040 ± 0.008 ^a, b^ | |
|  | **14** | 4.1 ± 0.7 ^a^ | 4.6 ± 0.6 ^a^ | | 0.022 ± 0.003 ^a^ | 0.039 ± 0.007 ^a, b^ | |
|  | **28** | 3.5 ± 1.1 | 3.3 ± 1.8 | | 0.019 ± 0.003 ^a^ | 0.022 ± 0.016 ^a^ | |
| **SD** |  | 1.1 ± 0.2 | 0.9 ± 0.4 | | 0.012 ± 0.002 | 0.010 ± 0.002 | |
| **HFD** | **60** | 0.4 ± 0.2 ^a^ | 0.6 ± 0.2 ^a^ | | 0.009 ± 0.001 ^a^ | 0.013 ± 0.003 ^a^ | |
|  | **90** | 0.5 ± 0.1 ^a^ | 0.5 ± 0.1 ^a^ | | 0.011 ± 0.002 | 0.012 ± 0.002 | |

Data are mean ± SD (n=8-10). Statistical significance was determined by ANOVA and Bonferroni post-test. ^a^*P*<0.05 *versus* its corresponding SD; ^b^*P*<0.05 *versus* male under the same diet conditions.
