# Supplementary material for: Hypothalamic endocannabinoids inversely correlate with the development of diet-induced obesity in male and female mice
Source: J Lipid Res. 2019 May 28;60(7):1260–9. doi: 10.1194/jlr.M092742 (PMC6602126; doi:10.1194/jlr.M092742)
Supplement: Supplemental Data [file 10.1194_M092742_jlr.M092742-8.docx]

**Supplemental Table S.3.** Plasmatic endocannabinoids levels (ng/mL plasma).

|  | | **2-AG** | | **AEA** | |
| --- | --- | --- | --- | --- | --- |
| ***Days on diet*** | | ***Male*** | ***Female*** | ***Male*** | ***Female*** |
| **SD** |  | 18.9 ± 2.3 | 22.0 ± 6.3 | 1.15 ± 0.09 | 2.0 ± 0.4 ^b^ |
| **HFD** | **7** | 19 ± 5.8 | 22.9 ± 6.3 | 1.01 ± 0.02 | 1.2 ± 0.2 |
|  | **14** | 19 ± 10 | 24.0 ± 9.6 | 1.01 ± 0.03 | 1.3 ± 0.3 |
|  | **28** | 22.2 ± 6.4 | 36.0 ± 12.6 | 1.13 ± 0.08 | 2.2 ± 0.6 ^b^ |
| **SD** |  | 6.3 ± 3.4 | 3.9 ± 0.9 | 1.9 ± 0.1 | 1.4 ± 0.6 |
| **HFD** | **60** | 10.8 ± 2.0 ^a^ | 9.8 ± 2.3 ^a^ | 2.2 ± 0.4 | 2.7 ± 0.6 ^a^ |
|  | **90** | 10.7 ± 1.5 ^a^ | 13.2 ± 3.6 ^a^ | 2.0 ± 0.3 | 4.0 ± 1.0 ^a, b^ |

Data are mean ± SD (n=8-10). Statistical significance was determined by ANOVA and Bonferroni post-test. ^a^*P*<0.05 *versus* its corresponding SD; ^b^*P*<0.05 *versus* male under the same diet conditions.
